# Unlocking the Potential of Dental-Derived Mesenchymal Stem Cells in Regenerative Medicine

**DOI:** 10.3390/jcm12113804

**Published:** 2023-06-01

**Authors:** Marco Tatullo, Sandro Rengo, Gilberto Sammartino, Gaetano Marenzi

**Affiliations:** 1 Department of Translational Biomedicine and Neuroscience (DiBraiN), University of Bari Aldo Moro, 70124 Bari, Italy; 2 Honorary Senior Clinical Lecturer, University of Dundee, Dundee DD1 4HR, UK; 3 MIRROR—Medical Institute for Regeneration and Repairing and Organ Replacement, Interdepartmental Center, School of Medicine, University of Bari Aldo Moro, 70124 Bari, Italy; 4 Department of Neurosciences, Reproductive and Odontostomatological Sciences, Postgraduate School of Oral Surgery, University “Federico II” of Naples, 80131 Naples, Italy; sandro.rengo@unina.it (S.R.); gilberto.sammartino@unina.it (G.S.); gaetano.marenzi@unina.it (G.M.)

## 1. Introduction

Over the past few decades, life expectancy has been increasing in several countries. However, this positive trend is accompanied by a rise in the occurrence of inflammatory and degenerative diseases, including neurodegenerative conditions, autoimmune diseases, metabolic disorders, and cancers.

Within this context, regenerative medicine emerges as an interdisciplinary field that integrates two essential areas: research and clinical practice, aiming to promote the regeneration and restoration of tissues and organs and additionally restore their physiological conditions. Specifically, regenerative medicine is based on an understanding of the mechanistic pathways that drive cellular and tissue regeneration, along with exogenous and endogenous factors. This knowledge is used to develop new therapeutic options for regenerating or restoring the functions of tissues and organs damaged by various conditions [1].

Tissue engineering has developed with an increased impact on medical procedures, involving last-generation biomaterials, newly discovered stem cell lines, and biologically active substances (e.g., MiRNA or Circular-RNA), to aid the physiological processes leading to tissue repair. Over the years, researched have developed various types of scaffolds aiming to increase their biomimetic ability; scaffold functionalization has been obtained with different degrees of porosity (to facilitate cell growth, nutrient transport, and waste removal), biodegradability, biocompatibility, mechanical properties, and physical behavior.

Currently, novel biomaterials are designed for long-term contact with biological tissues, and they can simultaneously act as supportive and bioactive scaffolds [1,2,3]. These biomimetic materials interact with the surrounding tissues through molecular recognition mechanisms. One of the main challenges in regenerative medicine is the design of three-dimensional scaffolds that actively interact with different cells, promoting in situ tissue regeneration. Stem cells, with their ability to differentiate into various cell types, play a crucial role in this field [1,2]. Mesenchymal stem cells (MSCs) have been extensively studied for their potential roles in tissue homeostasis, tissue engineering, and regenerative medicine. They exhibit several properties, such as capacities for modulating inflammation, promoting angiogenesis, reducing tissue fibrosis, exerting immunosuppressive effects, and multipotential differentiation [1,2].

MSCs can be sourced from various tissues and organs, including bone marrow, adipose tissue, the periosteum, umbilical cord, placenta, trabecular bone, skeletal muscle, and oral cavity [3]. Oral-derived mesenchymal stem cells (oral MSCs) have attracted growing attention in the scientific community due to their unique characteristics; in fact, in addition to possessing multipotency similar to other MSCs, oral MSCs have the advantages of easy accessibility, genomic stability, and a faster proliferation rate compared to bone marrow-derived MSCs [3,4,5,6,7,8].

The main types of oral MSCs include dental pulp stem cells (DPSCs), stem cells from human exfoliated deciduous teeth (SHEDs), periodontal ligament stem cells (PDLSCs), dental follicle stem cells (DFSCs), apical papilla stem cells (SCAPs), gingival stem cells (GMSCs), and human periapical cyst stem cells (hPCy-MSCs) [4].

Interestingly, human periodontal ligament stem cells (hPDLSCs) not only possess wide-ranging differentiation capabilities but also exhibit immunosuppressive effects [5]. Specifically, hPDLSCs have been shown to inhibit the proliferation of B and T lymphocytes [6] and shift macrophage polarization toward the M2 phenotype [7]. These characteristics contribute to tissue homeostasis, direct the inflammatory reaction towards the healing phase, and have potential therapeutic implications [8].

Furthermore, the concept of obtaining MSCs from “biological waste” has gained significant attention in the scientific community. This concept has been translated into the isolation of MSCs from inflamed human dental cysts (hPCy-MSCs); numerous studies have demonstrated the great clinical potential of hPCy-MSCs, particularly in bone regeneration and in the production of organoids, such as the midbrain-like organoids mimicking the deficit in dopaminergic neurons involved in neurodegenerative disorders including Parkinson’s disease (PD) [9].

Dentin-derived MSCs, originating from the neural crest, exhibit notable neuro-regenerative potential, making them attractive for the treatment of neurodegenerative disorders. These cells express neuronal markers under basal conditions, such as nestin, beta-3-tubulin, receptors for neurotrophins, and neurofilaments [10,11].

Several research groups are actively involved in the field of structural and functional regeneration of bone and dental tissue, which represents an intriguing and extensively debated topic in medicine. Beyond dentistry, oral MSCs have been combined with innovative biotechnologies in the fields of bone regeneration. In fact, bone defects arising from trauma, disease, surgery, or congenital malformations pose significant global health challenges [12,13]. In this context, ascorbic acid (AA), commonly known as vitamin C, emerges as a prominent water-soluble nonenzymatic antioxidant that exerts regulatory effects on bone cell metabolism and vitality [12]. In vitro studies have demonstrated that AA has the ability to diminish osteoclast activity and to expedite bone healing by promoting the osteogenic differentiation of MSCs [12].

MSC-based therapy for bone and cartilage repair [14] currently includes several long-term approaches used to address tissue damage and slow down disease progression. While pharmacological and regenerative therapies have shown promising results, extensive studies are underway to evaluate strategies ensuring the greater efficacy and reduced side effects of such protocols [15].

## 2. Conclusions

Extensive research conducted in both preclinical and clinical settings has provided valuable insight into the crucial role played by mesenchymal stem cells (MSCs) in maintaining tissue homeostasis. MSCs have demonstrated immense potential in tissue engineering and regenerative medicine. Oral MSCs have the further and unique characteristics, being easily obtainable from several sources with interesting differentiating behaviors, thus making them promising candidates for a wide range of clinical applications.

Looking to the future, ongoing investigations will play a critical role in overcoming the existing limitations of regenerative medicine. Advancements in our understanding of the precise mechanisms through which oral MSCs exert their overall therapeutic effects will pave the way for the development of innovative strategies. Additionally, through exploring the potential of oral MSCs in combination with other biotechnologies, these cells will contribute significantly to the development of personalized regenerative therapies.
